# The RESPECT study: a feasibility randomised controlled trial of a sexual health promotion intervention for people with serious mental illness in community mental health services in the UK

**DOI:** 10.1186/s12889-020-09661-x

**Published:** 2020-11-17

**Authors:** Elizabeth Hughes, Natasha Mitchell, Samantha Gascoyne, Thirimon Moe-Byrne, Amanda Edmondson, Elizabeth Coleman, Lottie Millett, Shehzad Ali, Francine Cournos, Ceri Dare, Catherine Hewitt, Sonia Johnson, Harminder Dosanjh Kaur, Karen McKinnon, Catherine Mercer, Fiona Nolan, Charlotte Walker, Milton Wainberg, Judith Watson

**Affiliations:** 1 School of Healthcare, Faculty of Medicine and Health, University of Leeds, Leeds, UK; 2 Centre for Applied Research in Health, School of Human and Health Sciences, University of Huddersfield, Huddersfield, England; 3 Department of Health Sciences, University of York, York, England; 4 Division of Psychiatry, University College London, London, England; 5 New York State Psychiatric Institute and Columbia University, New York, USA; 6 Experts by experience, England; 7 Camden and Islington NHS Foundation Trust, England; 8 Institute for Global Health, University College London, London, England; 9 School of Health and Social Care, University of Essex, Colchester, England

**Keywords:** Sexual health, Sexual behavior, Mental health, Psychosis, Feasibility, Randomised controlled trial

## Abstract

**Background:**

People with serious mental illness (SMI) have sexual health needs but there is little evidence to inform effective interventions to address them. In fact, there are few studies that have addressed this topic for people with SMI outside USA and Brazil. Therefore, the aim of the study was to establish the acceptability and feasibility of a trial of a sexual health promotion intervention for people with SMI in the UK.

**Method:**

The RESPECT study was a two-armed randomised controlled, open feasibility trial (RCT) comparing Sexual health promotion intervention (3 individual sessions of 1 h) (I) or treatment as usual (TAU) for adults aged 18 or over, with SMI, within community mental health services in four UK cities. The main outcome of interest was the percentage who consented to participate, and retained in each arm of the trial, retention for the intervention, and completeness of data collection. A nested qualitative study obtained the views of participants regarding the acceptability of the study using individual telephone interviews conducted by lived experience researchers.

**Results:**

Of a target sample of 100, a total of 72 people were enrolled in the trial over 12 months. Recruitment in the initial months was low and so an extension was granted. However this extension meant that the later recruited participants would only be followed up to the 3 month point. There was good retention in the intervention and the study as a whole; 77.8% of those allocated to intervention (*n* = 28) received it. At three months, 81.9% (30 I; 29 TAU) and at 6 months, 76.3% (13 I and 16 TAU) completed the follow-up data collection. No adverse events were reported. There was good completeness of the data. The sexual health outcomes for the intervention group changed in favour of the intervention. Based on analysis of the qualitative interviews, the methods of recruitment, the quality of the participant information, the data collection, and the intervention were deemed to be acceptable to the participants (*n* = 22).

**Conclusions:**

The target of 100 participants was not achieved within the study’s timescale. However, effective strategies were identified that improved recruitment in the final few months. Retention rates and completeness of data in both groups indicate that it is acceptable and feasible to undertake a study promoting sexual health for people with SMI. A fully powered RCT is required to establish effectiveness of the intervention in adoption of safer sex.

**Study registration:**

**ISRCTN Registry** ISRCTN15747739 prospectively registered 5th July 2016.

## Introduction

People who live with serious mental health illness (SMI) are sexually active [1]; and some engage in sexual risk behaviour (such as condom-less sex) [2]. This may explain why people with SMI are at increased risk of Human Immunodeficiency Virus (HIV) infection [3], sexually transmitted infections [4], unintended pregnancy and abortion [5] compared to the general population. The reasons for engaging in sexual risk behaviour may include hyper-sexuality when in an acute phase of illness; co-occurring drug and alcohol problems, and being vulnerable to sexual coercion, exploitation and abuse [6]. Despite these concerns, there has been a lack of attention to sexual health promotion in mental health care settings including staff avoiding the topic of sex and reporting significant structural and personal barriers to having conversations about sexual health [7–9]. These barriers include lack of knowledge about sexual health and sexual health services, concerns about the topic causing embarrassment or distress, and a lack of local or national policy drivers.

A number of studies have been conducted in the USA [10] which sought to evaluate tailored sexual health interventions for people with serious mental illness. These studies were randomized trials of group interventions (compared with treatment as usual or other time and attention control) and were targeted at HIV risk behaviour for people who have significant and long term mental health problems. The interventions ranged from brief 2–3 sessions [11] to a more intensive 12 session intervention which was for people who were homeless and had mental illness [12]. These studies have shown promise in the in terms of engaging and retaining people with SMI in the interventions, but have not always demonstrated an impact on adoption of safer sexual practices (such as increased use of condoms) [13].

The National Institute for Health Research (NIHR) commissioned a feasibility study (HTA 14/172/01) to develop and evaluate a bespoke sexual health intervention targeted at those with SMI. As there had been no previous trials of this nature in the UK, the first step in the process for evaluation of a complex intervention [14] is to assess feasibility and acceptability in order to establish the parameters for a fully powered trial.

## Methods

### Design

The RESPECT study was a pragmatic, multi-centred, open feasibility randomised controlled trial (RCT). Participants meeting the eligibility criteria were individually randomised (1:1) to receive either:

- The control arm: treatment as usual (TAU) which consisted of usual mental health care. All participants were free to pursue reproductive health and sexual health services via general services in their local area.
- The intervention arm: in addition to TAU, participants took part in three sessions of sexual health promotion, each of 1 h.

Irrespective of arm of the trial, all participants received written information on local sexual health, contraceptive services, some condoms, and national helplines at the baseline appointment.

### Setting

The study took place in the National Health Service (NHS) community mental health services which provides mental health support to people with severe mental illness who live in the community. People in receipt of this service will have a named care coordinator (often a mental health nurse or social worker) and will see their care coordinator as well as other support staff as well as regular reviews by other members of the multi-disciplinary team including psychiatrist.

### Sample size

The sample size calculations were based on estimating attrition rates and standard deviation of the primary outcome. Assuming 30% of participants were lost to follow up (as in the SCIMITAR pilot trial [15]) with a sample size of 100, then the 95% confidence interval for this level of attrition would be the observed difference ± 9 percentage points (i.e. between 21 and 39% [16];). Hence an external pilot trial of 100 participants would ensure robust estimates of follow-up in this population. Furthermore, an external feasibility study of at least 70 measured subjects provides robust estimates of the standard deviation of the outcome measure to inform the sample size calculation for the subsequent larger definitive fully powered trial.^11^

### Recruitment

#### Participant eligibility

##### Inclusion criteria

- people on the case load of selected community mental health services within each NHS site;
- diagnosed with a “severe mental illness” (defined as schizophrenia, other psychosis, bipolar affective disorder, schizoaffective disorder, major depressive disorder);
- aged 18 and over;
- willing and able to provide written informed consent.

##### Exclusion criteria

- having an acute exacerbation of their mental illness that precluded them from active participation (as indicated by hospitalisation and/or being under the crisis/home treatment team at the time of consenting);
- having a case note diagnosis that did not meet the criteria of SMI (see inclusion);
- having a severe physical illness that precluded them from active participation;
- a significant cognitive impairment (such as an organic brain disorder) as determined by case notes;
- a non-English speaker (adapting the intervention is currently beyond the scope of this study);
- lacking capacity to consent (as guided by the Mental Capacity Act 2005);
- being unable or unwilling to give written informed consent;
- being on the Sex Offenders Register, or having a history of inappropriate sexual behavior*.

*as reported by the care coordinator at the time of screening. “Inappropriate sexual behavior” was deemed to where the person has a known history of sexualized conversations or touching that would be uncomfortable or distressing for the researchers.

All case managers in the selected community mental health teams (CMHTs) were informed of the inclusion and exclusion criteria, and were contacted regarding potential participants to check that there were no areas for concern or researcher safety (such as regarding safety of home visits) prior to entry into the study.

### Recruitment into the trial

Potentially eligible participants were identified using three main methods: screening of caseloads of community mental health staff for potentially eligible people; direct approach to people using mental health services by research staff in clinic waiting rooms, and self-referral (via study email, telephone or via an online form on the study website). The details for self-referral were provided on all participant-facing materials such as the posters and leaflets.

### Flow of participants from identification to entry into study

The numbers of people who were screened, eligible and consented to participate were recorded where possible. Eligible patients who did not wish to take part (i.e. unwilling to give consent) and those found to be ineligible went on to receive usual care from the service without prejudice.

### Informed consent and baseline assessment

Once eligibility was confirmed by mental health service, a RESPECT researcher arranged a convenient time and venue to meet with the potential participant to discuss participation. The first part of the meeting involved the researcher fully explaining the study and what would be involved (as per information sheet) and an opportunity for the person to ask questions and seek clarification. Written informed consent was then obtained and baseline data was collected (or a further date was arranged for baseline data collection). Participants received a £10 voucher for baseline, and for each follow-up data collection point as a token of gratitude for participating.

### Trial intervention

The overall aim of the intervention was for people to adopt safer sexual behaviours (as evidenced by reduced condom-less sex) and engage in more positive sexual relationships. The intervention was based on the Information Motivation Behaviour (IMB) model of sexual health behaviour change [17] and ensured that it addressed the following:

1. Addressing any information needs using quizzes and exercises;
2. Increase motivation to adopt safer sexual behaviours using exercises and conversations;
3. Increase behavioural skills (and self-efficacy) to adopt safer sexual behaviours through role play and skills practice.

The intervention was designed to be delivered over 3 × 1-h sessions that were delivered face-to-face by a specifically trained mental health worker. These interventionists were identified within each site and received training and an intervention pack prior to being allocated to participants. The sessions could be delivered at the local clinical service (where the person usually attended) or at their homes.

The manual was developed by an Intervention Mapping process [18] using a combination of review of existing manuals that had been developed specifically for people with serious mental illness as well as consultation with service users and other stakeholders. Attention was paid to addressing the knowledge, motivational and behavioural and social skills deficits that have been identified as challenges to adopting safer sexual behaviours in this group [6]. Iterations of the manual were reviewed by stakeholders and the members of the RESPECT study Patient and Public Involvement (PPI) representatives and the content and format was refined based on feedback and discussion. The development of the intervention is described in more detail in the published NIHR final report [19]. The delivery of the topics was designed to be interactive and used a series of quizzes, exercises and scenarios to generate discussion. The aim of the exercises within the sessions was to facilitate discussion about knowledge about sexual health and to supplement the gaps in knowledge in the session or signpost people to local sexual health and family planning services. In terms of the theoretical underpinning of the intervention (The IMB model) the quizzes were designed to improve knowledge and the discussions related to own risks and choices was designed to promote the importance of considering changing behaviour (build motivation). In addition to developing a sense of importance of change, the intervention used exercises and role play to increase a sense of self-efficacy and self-worth. The role plays of negotiation and assertiveness skills as well as the practice of putting a condom on and off safely improved behavioural skills. All participants were offered condoms and sachets of lubricant at each session.

Summary of content:

- Session 1: Knowledge regarding safer sex including HIV and sexually transmitted infection quizzes; condoms and contraception and where to seek help and advice
- Session 2: Risky and less risky sexual behaviours for HIV; pros and cons of condom use; behavioural skills of using condoms (using a plastic condom demonstrator); contingency planning for risky sexual situations
- Session 3: Focus on relationships- signs of good and less good aspects of relationships; assertive communication; negotiating own needs and wishes in sexual relationships; developing an action plan for the future.

The intervention was delivered by a mental health worker from the NHS trust. They volunteered to support the study and were provided with 1 day training on how to deliver the intervention facilitated by the Chief Investigator (Hughes), and an accompanying intervention manual and pack containing all the materials needed to deliver it (copies of the manual are available by request from lead author).

### Control arm

Participants randomised to receive TAU continued to receive their usual care. TAU for sexual health (including contraception) included the freedom to access their local primary care and/or specialist sexual health services. Participants in the intervention and control arm were offered condoms and lubricant sachets as well as a localized list of sexual health services at baseline and follow-up appointments.

### Outcomes

The main outcome of the RESPECT study was to establish the feasibility and acceptability of an evidence-based intervention to promote sexual health, and to establish key parameters to inform a future main trial. In conjunction with the qualitative study, this was to be established by measuring recruitment rates, retentions rates and follow up completion rates.

### Secondary outcome assessment

The following outcome measures were collected at baseline, 3 months post randomisation and 6 months post randomisation:

- Sexual Risk Behaviour Assessment Schedule (SERBAS) [12]: a validated HIV risk behaviour measure which was developed in the USA, and has been validated for use with populations who have serious mental illness. It gathers information on sexual activity in the last 3 months and records frequency of high-risk behaviours (for HIV infection) such as intercourse without a condom, sexual activity under influence of drugs and alcohol, and sex work/sex trading. It takes into account sexuality and gender within the schedule.
- The National Survey of Sexual Attitudes and Lifestyle (Natsal) [20]: We have included specific items which cover broader aspects of sexual health including contraception use, STI and HIV tests, and knowledge on family planning advice.
- Knowledge about HIV (HIV-KQ) [21] a 17 item measure that assess’ knowledge about HIV (*This originally contained 18 item but we removed one question about lambskin condoms as these are no longer in use)
- Motivations to Engage in Safer Sex [21] is a 4 item scale to assess people’s own perception of their risk of infection with sexually transmitted infections
- Condom Self-efficacy Scale [21]: an 18 item Likert scale to assess attitudes towards the use of condoms as well as questions on self-efficacy in the use and negotiation of use.
- Behavioural Intentions for Safer Sex [21]: a six-item measure where patients are presented with a scenario describing a possible sexual encounter and asked to rate how likely it was that they would engage in six risky or protective behaviours (e.g., “I will tell the person I don’t want to have sex without a condom”). Patients responded to each behaviour using a 6-point scale (ranging from 0 definitely will not do to 5 definitely will do).
- Mental illness stigma scale (MISS-Q) [22]: a 32 item tool that has been developed and validated to measure a person’s perceived stigma as a result of their mental health problem and its impact on perceptions of attractiveness and opportunities for intimate relationships.
- EQ-5D-5L (EuroQol): a standardised instrument for use as a measure of health outcome applicable to a wide range of health conditions and treatments (https://euroqol.org) (Licence permission to use in Supplementary materials).
- The Alcohol, Smoking and Substance Involvement Screening Test (ASSIST) [23]: developed for the World Health Organisation (WHO) by an international group of substance abuse researchers to detect and manage substance use and related problems in primary and general medical care settings.
- Recovering Quality of Life (ReQoL) [24]: a new 20 item patient reported outcome measure that has been developed to assess the quality of life for people with different mental health conditions.
- Cost assessment: Commonly used generic instruments to measure health-related quality of life (such as EQ-5D-5L) were used and assessed for completion rates at various time points and patterns of missing data. Sensitivity of generic instruments were evaluated against sexual health-specific clinical outcomes. A bespoke resource use questionnaire was designed to identify the key cost drivers and can be seen in the study report [19].

### Randomisation

Randomisation was performed by a secure, remote, telephone service based at York Trials Unit. An independent statistician at the University of York undertook the generation of the randomisation sequence. Randomisation was on a 1:1 basis using stratified block randomisation with stratification by centre and variable block sizes. Periodic checks were made on the computerised randomisation system during the trial following standard operating procedures:

- **Allocation concealment**: Randomisation was done by the researcher calling an independent person at the York Trials Unit who entered participant details into the trial database and the random allocation for that person was generated.
- **Sequence generator**: The randomisation was stratified by study site to ensure that the balance of allocation to intervention was evenly spread.
- **Blinding:** Participants were randomised into the study following completion of baseline data. Therefore, at baseline the researcher and the participant were blinded to the arm of the trial they would be allocated to. However due to the nature of a behavioural intervention compared with treatment as usual, it was not possible for the researcher or participant to be blinded at follow-up data collection.

### Trial completion and exit

Participants were considered to have exited the trial when they:

- withdrew consent
- had been withdrawn by interventionist/researcher for reasons of risk or harm to self and/or others
- had reached the end of the trial
- died

### Withdrawals

Withdrawal could occur at any point during the study at the request of the participant. When a participant expressed that they wished to withdraw from the study, a researcher would speak to that person to clarify the level of withdrawal. If the participant requested to be withdrawn from the intervention only, follow up data continued to be collected. All data were retained for all participants until the date of withdrawal unless they specifically requested that this be destroyed.

A participant could also be withdrawn without their consent from the intervention and/or the trial for reasons of risk or harm to self and/or others. This was only actioned where there was evidence of serious and significant risk and in accordance with the trial risk protocol.

### Adverse events (AE)

Adverse events were monitored by an independent Data Monitoring and Ethics Committee and the Trial Steering Committee (TSC). The DMEC/TSC would immediately be notified and asked to review any reported serious adverse events (SAEs) that were deemed to be study and/or intervention related.

### Statistical analysis

As this was a feasibility trial, no formal analysis was undertaken, and all analysis was descriptive. The flow of participants is detailed in a CONSORT flow diagram. The number of people screened, randomly assigned, receiving the intervention and providing outcome data is summarised overall and by trial arm. The number of individuals withdrawing from the intervention and/or the trial and any reasons for withdrawal is summarised by trial arm. To quantify the acceptability of the intervention the number of sessions attended is also summarised. All data is presented descriptively with no formal statistical analyses undertaken. For each data collection point and outcome measure, the numbers of non-responders is calculated and completion rates compared. The average caseload per therapist is detailed.

### Health economics

Economic analysis was conducted with the aim to evaluate the feasibility of collecting data on costs and health-related quality of life outcome from the UK health services perspective. Resource use data were collected to estimate: i) cost of delivering the intervention; and ii) individual-level cost of health service resource use by trial participants over the trial follow-up period of 6 months.

Finally, analysis of the cost and health-related quality of life data was conducted in terms of the overall response rate for each questionnaire, rate of missing items within each questionnaire as well as changes from baseline to follow-up in health service resource use as well as quality of life by treatment arm.

In addition a nested qualitative study was undertaken with a sub-sample of participants at the end of the study obtain qualitative data on the experience of being part of the RESPECT study. Participants had given consent at the start of the study to be re-contacted to be invited to take part in individual interviews conducted by phone. They were not interviewed by the same person who had collected the baseline and follow-up data to avoid social desirability responses. Lived experience researchers were involved in this aspect of the study along with the two main researchers. Interviews were digitally recorded and transcribed and then coded using thematic analysis. This nested study is described in more detail in the NIHR report [19] and also in a paper in preparation (please contact corresponding author for details).

## Results

**Fig. 1:**
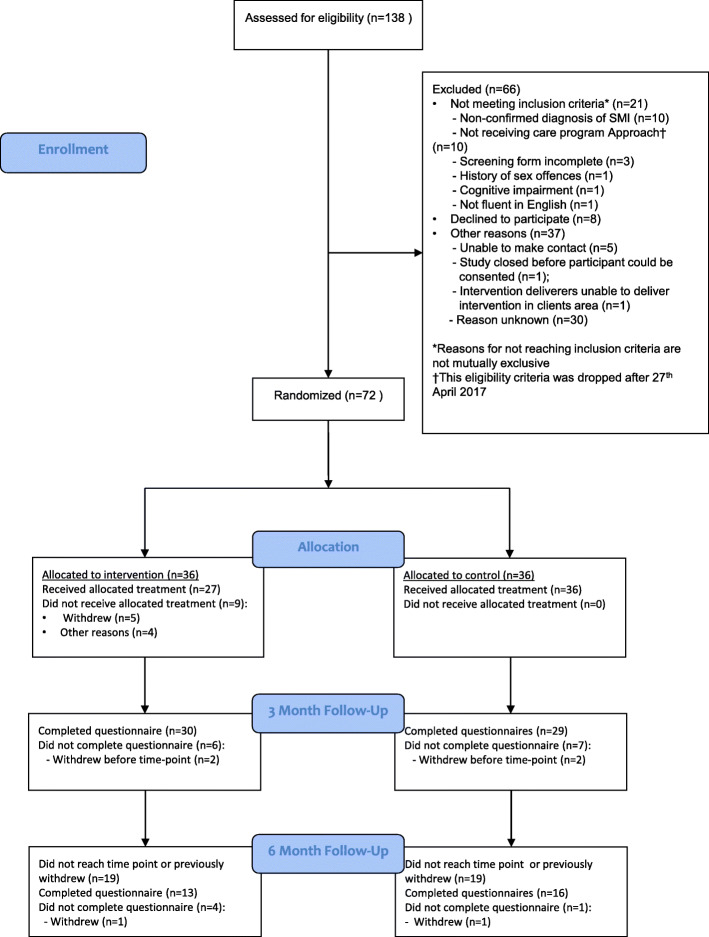
Consort Diagram

The flow of participants through the trial is detailed in the CONSORT flow diagram (Fig. 1). The number of people screened, randomly assigned, receiving the intervention, completing the study protocol and providing outcome data are summarised overall and by trial arm. The number of individuals withdrawing from the intervention and/or the trial and any reasons for withdrawal are summarised by trial arm. To quantify the acceptability of the intervention the number of sessions attended is also summarised. All data is presented descriptively with no formal statistical analyses undertaken. For each data collection point and outcome measure, the numbers of non-responders were calculated and completion rates were compared. The average caseload per therapist is detailed.

### Recruitment

**Fig. 2:**
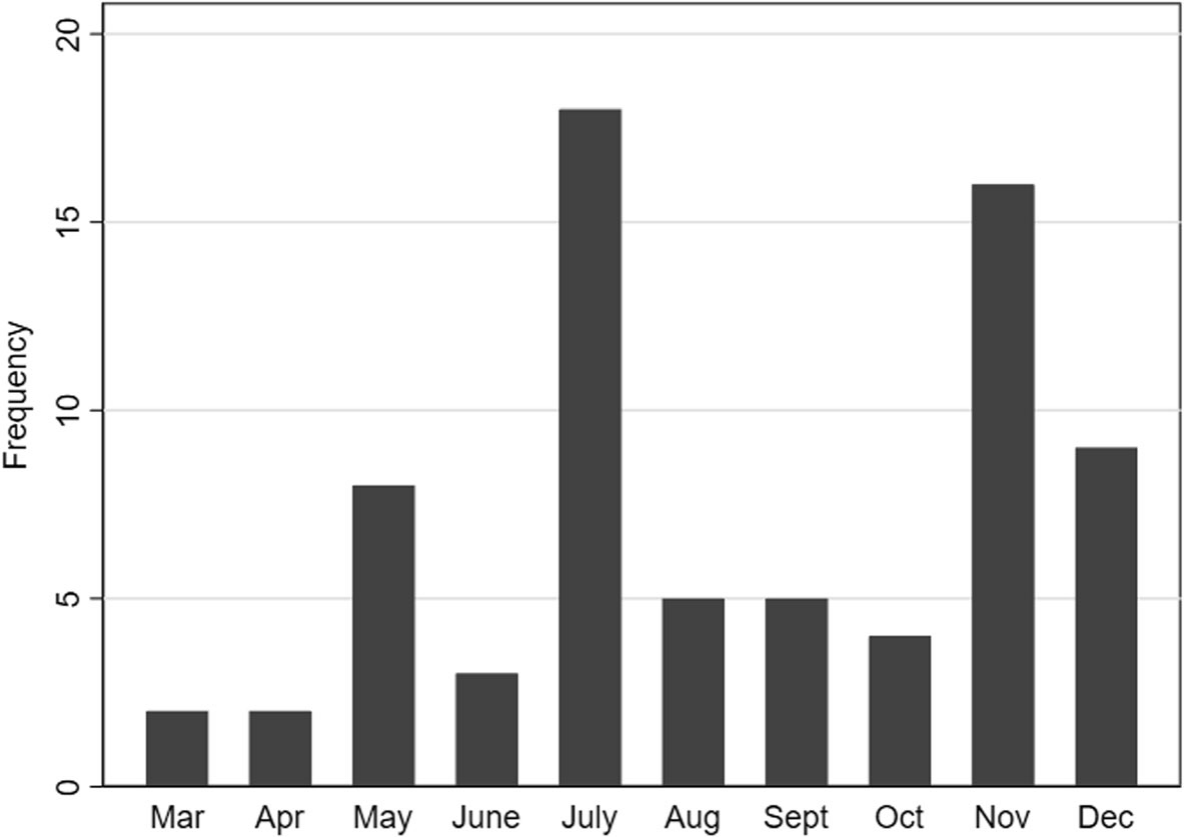
Recruitment

The original recruitment target was 100 people over 6 months. However, recruitment was slower than expected and changes to recruitment strategy were made in an attempt to increase recruitment after 3 months. This included focusing recruitment on a more direct service user approach (face to face, posting packs and follow-up phone calls). Following these changes, recruitment did improve (see Fig. 2).

Over the course of 12 months 138 people were recorded as being formally screened for eligibility. This number is based on data from the screening logs from the NHS Research and Development offices. However, it is likely the number that were eligible was much higher as many participants were notified about the study more informally via leaflets, posters and talking to their care coordinators. Of those formally screened, 117 (84.8%) met the eligibility defined for inclusion into the trial. This 84.8% eligible is much higher than the 50–60% anticipated based on previous studies [21, 25]. A total of 72 participants were randomised into the trial giving a recruitment rate of 61.5% (52.2% of screened participants then went on to enter the study), which was higher than the 40% which was predicted. The flow of participants can be seen in 2.

### Follow-up, withdrawals and intervention attendance

There was good retention in the trial. All participants were followed up at 3 month post randomisation, and a subsample (*n* = 38) were also followed up at 6 months (limited only due to time constraints of the end of the study period). At 3 months, 59 of the 72 participants completed the questionnaire (81.9%), split equally across the two arms (*n* = 30 intervention, and *n* = 29 control). Similarly, at 6 months 76.3% of participant due to complete the questionnaire did (*n* = 29, 13 intervention and 16 control). This shows that participants in both arms are willing to be involved and retain in the study, demonstrating that a future trial would have the ability to retain participants.

Overall, ten participants (13.9%) withdrew from the study. Two participants were withdrawn from follow-up only, one after discussion with the lead investigator and clinician (due to their poor mental state at the time of follow-up) and the other gave no reasons (one in each arm). Full withdrawal from the trial was requested by four participants; two in each treatment arm. One person changed their mind about taking part due to the topic, and a further three gave no reason.

Thirty-six participants (50.0%) were randomised to the intervention arm. The intervention was designed to consist of 31-hour sessions. Nine of these participants (25.0%) never started the intervention; five withdrew from treatment prior to starting. The first session was attended by 25 participants (69.4%), the second by 19 (52.8%) and the third by 18 (50.0%). However, several (*n* = 5) participants requested to combine sessions, so this might be an under-estimate of attendance. In total, 17 participants (47.2%) attended all three sessions and 22 participants were exposed to all the content (61.1% of those initially randomised; 81.5% of those who started the intervention). Table 1 presents the demographics of the participants in intervention and control arm.

**Table 1:** Participant Demographics

	Intervention (***n*** = 36)	Control (***n*** = 36)	Overall (***n*** = 72)
**Age**			
Mean (sd)	44.2 (12.1)	45.3 (11.5)	44.8 (11.8)
Median (min, max)	47.1 (22.9, 66.1)	46.9 (22.0, 65.1)	46.9 (22.0, 66.1)
**Gender, n (%)**			
Male	18 (50.0)	17 (47.2)	35 (48.6)
Female	17 (47.2)	17 (47.2)	34 (47.2)
Other	1 (2.8)	2 (5.6)	3 (4.2)
Missing	0 (0.0)	0 (0.0)	0 (0.0)
**Sexuality, n (%)**			
Heterosexual	29 (80.6)	30 (83.3)	59 (81.9)
Gay or lesbian	3 (8.3)	1 (2.8)	4 (5.6)
Bisexual	3 (8.3)	3 (8.3)	6 (8.3)
Prefer not to say	0 (0.0)	1 (2.8)	1 (1.4)
Other	1 (2.8)	1 (2.8)	2 (2.8)
**Ethnicity, n (%)**			
White British	23 (63.9)	23 (63.9)	46 (63.9)
White Irish	0 (0.0)	1 (2.8)	1 (1.4)
Black African	1 (2.8)	1 (2.8)	2 (2.8)
Black Caribbean	2 (5.6)	0 (0.0)	2 (2.8)
Black Other	0 (0.0)	1 (2.8)	1 (1.4)
Asian Indian	1 (2.8)	1 (2.8)	2 (2.8)
Asian Pakistani	1 (2.8)	2 (5.6)	3 (4.2)
Asian Bangladeshi	1 (2.8)	0 (0.0)	1 (1.4)
Asian Other	0 (0.0)	1 (2.8)	1 (1.4)
White & Black Caribbean	1 (2.8)	0 (0.0)	1 (1.4)
White & Asian	0 (0.0)	1 (2.8)	1 (1.4)
Other mixed background	1 (2.8	0 (0.0)	1 (1.4
Prefer not to say	1 (2.8)	1 (2.8)	2 (2.8)
Other	4 (11.1)	4 (11.1)	8 (11.1)
**Religion, n (%)**			
No religion	15 (41.7)	14 (38.9)	29 (40.3)
Muslim	3 (8.3)	3 (8.3)	6 (8.3)
Christian	14 (38.9)	13 (16.1)	27 (37.5)
Sikh	1 (2.8)	0 (0.0)	1 (1.4)
Buddhist	0 (0.0)	3 (0.0)	3 (4.2)
Hindu	0 (0.0)	0 (0.0)	0 (0.0)
Jewish	0 (0.0)	0 (0.0)	0 (0.0)
Prefer not to say	0 (0.0)	1 (2.8)	1 (1.4)
Other	1 (2.8)	1 (2.8)	2 (2.8)
Missing	2 (5.6)	1 (2.8)	3 (4.2)
**Highest qualification, (%)**			
None	3 (8.3)	3 (8.3)	6 (8.3)
GCSEs/GCEs/CSEs	9 (25.0)	2 (5.6)	11 (15.3)
AS/A Levels	6 (16.7)	6 (16.7)	12 (16.7)
Diploma	1 (2.8)	3 (8.3)	4 (4.6)
Higher Degree	7 (19.4)	5 (13.9)	12 (16.7)
Further Higher Degree	2 (5.6)	5 (13.9)	7 (9.7)
Vocational Education	4 (11.1)	6 (16.7)	10 (13.9)
Other	3 (8.3)	5 (13.9)	8 (11.1)
Missing	1 (2.8)	1 (2.8)	2 (2. 8)
**Employment, n (%)**			
Full time	1 (2.8)	5 (13.9)	6 (8.3)
Part time	4 (11.1)	3 (8.3)	7 (9.7)
Unable to work due to poor health	17 (47.2)	21 (58.3)	38 (52.8)
Unemployed	8 (22.2)	5 (13.9)	13 (18.1)
Retired	2 (5.6)	1 (2.8)	3 (4.2)
Student	2 (5.6)	1 (2.8)	3 (4.2)
Other	2 (5.6)	0 (0.0)	2 (2.8)
**Living arrangement, n (%)**			
Live with parent/career	4 (11.1)	7 (19.4)	11 (15.3)
Live alone	24 (66.7)	18 (50.0)	42 (58.3)
Live with relative	1 (2.8)	3 (8.3)	4 (5.6)
Live in a hostel	2 (5.6)	1 (2.8)	3 (4.2)
Live with a friend	2 (5.6)	2 (5.6)	4 (5.6)
With partner/spouse	2 (5.6)	4 (11.1)	6 (8.3)
Other	1 (2.8)	1 (2.8)	2 (2.8)
**Relationship status, n (%)**			
Single, not married	26 (72.2)	22 (61.1)	48 (66.7)
Married	2 (5.6)	5 (13.9)	7 (9.7)
Civil partnership	0 (0.0)	0 (0.0)	0 (0.0)
Co-habiting	2 (5.6)	0 (0.0)	2 (2.8)
In a relationship, not living together	4 (11.1)	6 (16.7)	10 (13.9)
Separated	0 (0.0)	1 (2.8)	1 (1.4)
Divorced	2 (5.6)	2 (5.6)	4 (5.6)
Widowed	0 (0.0)	0 (0.0)	0 (0.0)

### Sexual behaviour measure

The SERBAS asked participants to record the number of sexual acts, and those that were unprotected, that had been undertaken in the last 3 months. The percentage of total sex acts (oral, vaginal and anal) that were undertaken without protection is detailed by arm at each time point, by gender in Table 2.

**Table 2:** Percentage of total sex acts undertaken without protection (condom or other barrier)

Time point	Intervention		Control	
	Males	Females	Males	Females
Baseline	97	73	87	68
Month 3	85	59	78	75
Month 6	50	53	79	97

The number of participants in RESPECT was small, and 50% of the sample reported no sexual acts within the length of the study; this means that (like the reviews undertaken previously [10, 13]) there is no evidence in this study that the intervention has had a statistically significant effect reducing the number of sexual acts undertaken without protection. However, it can be seen from Table 2 that there does appear to be reduction in our population in those who received the intervention.

### Intervention delivery

There were 11 different interventionists who delivered the sessions. A total of 70 sessions were delivered. This gives an average of 6.4 sessions per therapist however there was wide range from 1 session to 28 sessions delivered per interventionist.. On average the sessions were 58 min long (excluding the combined sessions) and had been designed to be approximately 60 min long.

### Health economics

Unit costs of health service use were obtained from the UK national database of reference costs Department of Health [26] [ref], and the Unit Costs of Health and Social Care report produced by the Personal and Social Services Resource Unit [ref] [27].

### Qualitative feedback about the experience of participation

A sub-sample of 22 people (in control and intervention arms) were interviewed.

The results of the qualitative study are reported in more detail in the NIHR report [19] but in summary the participants were very positive about the whole experience of taking part in RESPECT. There was no overall preference for any one method of recruitment, but there was a common theme of stating a preference in being able to have a conversation with someone about participating (such as with their care coordinator). This is interesting to note considering care coordinators had not engaged directly conversations about the study during recruitment and the more successful route had been by direct contact with potential participants. The study information provided was reported to be easy to understand and provided enough information to prepare them for what would be involved in taking part. People found the data collection comfortable and not distressing despite the fact that for some people the data collection appointments could be up to 2 h and involve questions about sexual activity. They reported that the research staff were friendly and approachable and they valued flexibility in times and locations of appointments. They appreciated the voucher as a “thank you” for taking part. Some felt that parts of the data collection was a bit boring and repetitive, but not to the point that it was very uncomfortable. For those who received the intervention, they found it interesting, thought provoking and informative. They liked the interactive nature of the sessions and again there were comments appreciating the flexibility of times and locations of delivery. Some of the participants mentioned they had never spoken to a care coordinator about sexual health before, and one participant reported that taking part in RESPECT had been an “ice breaker” meaning that they had begun to have conversations with their care coordinator about the subject. Finally, almost all of the 22 participants said they would recommend participating in the study to other people.

## Discussion

The RESPECT study was the first study to test the feasibility of undertaking a randomised trial of a sexual health intervention outside the Americas. Few studies related to sex and sexuality have been conducted in the UK, and as well as identifying if such as study is acceptable and feasible, this also has provided useful data on how to recruit to and collect data on sexual health for people who use community mental health services.

As this was designed to establish feasibility, it was not powered to detect statistically differences between intervention and control group so despite the intervention group outcomes appearing to favour a benefit from the intervention, this has not been definitively established in this study. The time to recruit the sample was in hindsight underestimated. The target of 100 was not achieved, even with an extension to the recruitment phase. However the attrition was not as high as the predicted 30%. The other limitation is a lack of detail on exact numbers screened at each site, and how many of those eligible actually received information regarding the study. Therefore it is not known for sure if the lower recruitment was due to the study being unattractive to eligible participants, or if it was because the information did not reach the potential participants. Certainly, the recruitment did improve using a more direct approach rather than relying on busy mental health staff to discuss the study and pass on information. The participants who were interviewed did not express a preference for recruitment method but did feel that it was important to be able to speak to someone (such as their care coordinator about the study).

The profile of people recruited broadly reflect the characteristics of people with serious mental Illness (see Table 1). In addition to the demographics, the average ReQoL scores reflect those of a clinical population [24]. Equal numbers of men and women were recruited and evenly distributed across both arms. Retention was similar in both arms of the trial. The study was conducted over several services and a range of geographical areas in England therefore the challenges and solutions that have been identified are likely to be applicable to further sites in a larger trial.

The fact that 72 people with serious mental illness across several services in England engaged with the study is a positive finding. This indicates that it is feasible to engage people with SMI in a study related to sexual health without any adverse events. Retention was good in terms of both the data collection (both in control and intervention group) in spite of the fact that data collection appointments took between one and 2 h and focused on sexual behaviour. The intervention was well attended; most people who attended at least the first session of the intervention went on to complete all three. The feedback from the qualitative interviews comfirmed that this was perceived to be a comfortable and interesting study to participate in,

The feasibility study has identified a number of issues that could be addressed in a future fully powered trial of effectiveness: This includes dedicating more time to support the role of the care coordinators in community mental health teams in terms of promoting the topic and allaying any concerns regarding the study. In addition, there should be sufficient people trained and able to deliver the intervention within each service (and of both genders). There were periods in the study where there was a lack of availability of a trained interventionist, and some participants did not receive the intervention due to this delay.

Many people were not sexually active during the study period (even if they had been active in other periods) and so this means that the primary outcome of N% “condom less sex” could be problematic to base the sample size calculation for a future trial. However, the intervention sought to be broader than simply using condoms, and also includes the whole range of contraceptive choices, as well as assertiveness skills and planning within sexual relationships, in line with the World Health Organisation [28] definition. This sees sexual health as broader than simply the prevention of infections; and incorporates the right to express one’s own sexuality free from abuse and coercion. The RESPECT study gave people an opportunity to have frank discussions about their past current and future sexual encounters, as well as receiving a clear message that sexual expression is important part of being human and having a mental illness should not exclude them from what is actually a fundamental human right. One of the measures assessed behavioural intentions to adopt safer sex and at follow-up the scores were positive in the direction of the intervention. Therefore it will be important in a future trial that people are not excluded on the basis of not being currently sexually active, and the sample size will have to be larger to account for the fact that some people may not be having sex during the study period itself.

Despite not quite achieving the target sample, at the end of recruitment period there were other potential recruits identified, and the recruitment graph suggests that recruitment improved over time, so it is reasonable to assume that targets could be achieved in a future trial with sufficient sites fully engaged and with capacity to deliver on the intervention for the trial period.

## Conclusion

People with serious mental illness are interested in sexual health and have a range of sexual health needs that need exploring and responding to. This study was able to recruit a sample of people who are living with serious mental illness and retain them in both the intervention and data collection. The topic did not trigger distress or other harms. Therefore undertaking sexual health research with people with serious mental illness is important, and this study demonstrated that it is feasible, safe and acceptable to participants.

## Data Availability

Data and materials are available by contacting the lead author.

## Abbreviations

ASSIST: The Alcohol, smoking and substance involvement screening test
CMHT: Community mental health team
EQ5D-5 L: EuroQol instrument that measures quality of life
HIV: Human imunodeficiency virus
HIV-KQ: Human Immunodeficiency virus knowledge quiz
MISS-Q: Mental illness sexual stigma questionnaire
NATSAL: National survey of sexual attitudes and lifestyle
NHS: National health service
NIHR: National institute for health research
PPI: Public and patient involvement
RCT: Randomised Controlled Trial
REQoL: Recovering quality of life, measures quality of life for people with mental illness
SERBAS: Sexual risk behaviour assessment schedule
SMI: Serious mental illness
TAU: Treatment as usual
